# Di-μ-chlorido-bis­{[4-amino-3,5-bis­(2-pyrid­yl)-4*H*-1,2,4-triazole-κ*N* ^1^]chloridomercury(II)}

**DOI:** 10.1107/S1600536811029886

**Published:** 2011-08-02

**Authors:** Jian Guo, Min Tang, Jing Chen, Cheng-Peng Li

**Affiliations:** a College of Chemistry, Tianjin Key Laboratory of Structure and Performance of Functional Molecules, Tianjin Normal University, Tianjin 300387, People’s Republic of China

## Abstract

In the centrosymmetric binuclear title complex, [Hg_2_Cl_4_(C_12_H_10_N_6_)_2_], the Hg^II^ ion is five-coordinated by two N atoms and three chloride ions with a distorted square-pyramidal geometry. In the complex, there is an intra­molecular N—H⋯N hydrogen bond. In the crystal, the binuclear units are connected by inter­molecular N—H⋯Cl hydrogen bonds, as well as π–π stacking inter­actions [centroid–centroid distances = 3.526 (2) and 3.696 (2) Å], forming a two-dimensional layered structure parallel to (010).

## Related literature

For background information on triazole derivatives, see: Klingele *et al.* (2009 ▶); Shao *et al.* (2004 ▶); Huang *et al.* (2011 ▶). For the coordination compounds synthesized with related triazole ligands, see: Du *et al.* (2007 ▶, 2008 ▶). For a description of the geometry of complexes with five-coordinate metal ions, see: Addison *et al.* (1984 ▶).
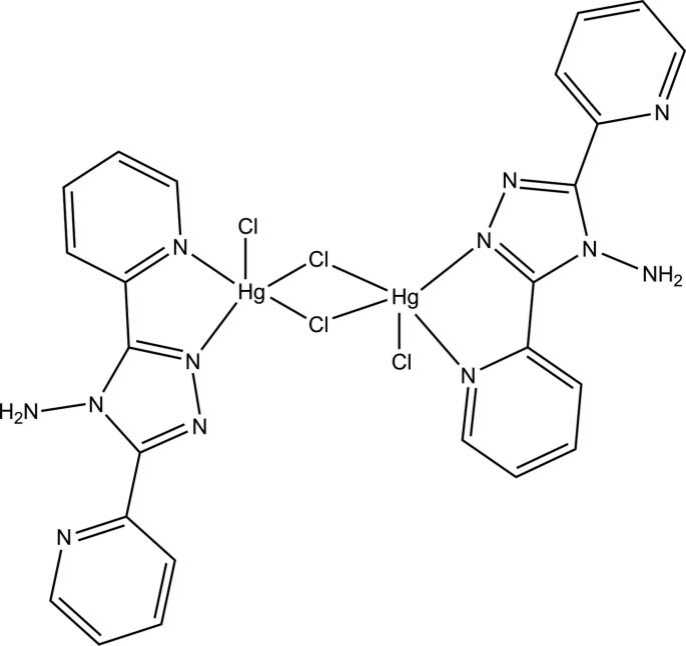


## Experimental

### 

#### Crystal data

[Hg_2_Cl_4_(C_12_H_10_N_6_)_2_]
*M* *_r_* = 1019.50
Orthorhombic, 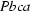

*a* = 11.3634 (4) Å
*b* = 14.9962 (6) Å
*c* = 17.2328 (7) Å
*V* = 2936.6 (2) Å^3^
*Z* = 4
Mo *K*α radiation
μ = 10.85 mm^−1^
*T* = 296 K
0.28 × 0.22 × 0.20 mm

#### Data collection

Bruker SMART CCD area-detector diffractometer
Absorption correction: multi-scan (*SADABS*; Sheldrick, 1996 ▶) *T*_min_ = 0.475, *T*_max_ = 1.000
14063 measured reflections
2596 independent reflections
2071 reflections with *I* > 2σ(*I*)
*R*_int_ = 0.023

#### Refinement

*R*[*F* ^2^ > 2σ(*F* ^2^)] = 0.022
*wR*(*F* ^2^) = 0.058
*S* = 1.08
2596 reflections
190 parameters
H-atom parameters constrained
Δρ_max_ = 0.53 e Å^−3^
Δρ_min_ = −1.26 e Å^−3^

### 

Data collection: *SMART* (Bruker, 2007 ▶); cell refinement: *SAINT* (Bruker, 2007 ▶); data reduction: *SAINT*; program(s) used to solve structure: *SHELXS97* (Sheldrick, 2008 ▶); program(s) used to refine structure: *SHELXL97* (Sheldrick, 2008 ▶); molecular graphics: *DIAMOND* (Brandenburg, 1999 ▶); software used to prepare material for publication: *SHELXTL* (Sheldrick, 2008 ▶).

## Supplementary Material

Crystal structure: contains datablock(s) I, global. DOI: 10.1107/S1600536811029886/su2294sup1.cif

Structure factors: contains datablock(s) I. DOI: 10.1107/S1600536811029886/su2294Isup2.hkl

Additional supplementary materials:  crystallographic information; 3D view; checkCIF report

## Figures and Tables

**Table 1:** Hydrogen-bond geometry (Å, °)

*D*—H⋯*A*	*D*—H	H⋯*A*	*D*⋯*A*	*D*—H⋯*A*
N5—H5*A*⋯Cl2^i^	0.89	2.80	3.446 (3)	131
N5—H5*A*⋯Cl1^ii^	0.89	2.77	3.512 (4)	142
N5—H5*B*⋯Cl1^iii^	0.89	2.62	3.452 (4)	155
N5—H5*B*⋯N6	0.89	2.47	2.956 (5)	115

Symmetry codes: (i) 
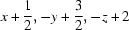
; (ii) 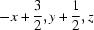; (iii) 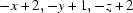.

**Fig. 1.**
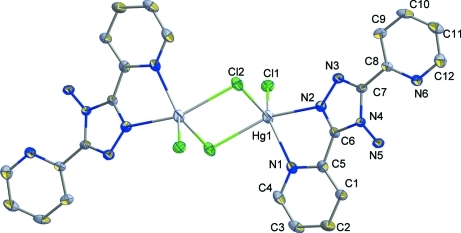
The molecular structure of the title compound showing the numbering scheme and displacement ellipsoids drawn at the 30% probability level.

**Fig. 2.**
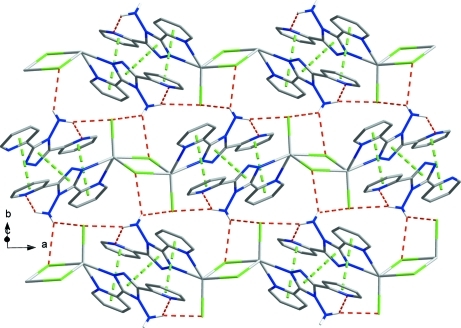
A view along the c-axis of the two-dimensional network in the crystal of the title compound, showing the N–H···N and N–H···Cl hydrogen bonds (red dashed lines) and the π–π stacking interactions (green dashed lines) [See Table 1 and the comment section for details].

## supplementary crystallographic information

### Comment

1,2,4-triazole and its derivatives have been extensively used to prepare diverse coordination complexes (Klingele *et al.*, 2009; Shao *et al.*, 2004; Huang *et al.*, 2011). Recently, a series of interesting metallosupramolecular systems have been constructed using the triazole ligands 4-amino-3,5-bis(4-pyridyl)-1,2,4-triazole and 4-amino-3,5-bis(3-pyridyl)-1,2,4-triazole, using different synthetic methods (Du *et al.*, 2007, 2008). In this context, the analogous ligand, 4-amino-3,5-bis(2-pyridyl)-1,2,4-triazole (2-bpt), that may exhibit different conformations and coordination modes, has received our attention.

Herein, we present the title binuclear complex obtained by the reaction of 2-bpt and HgCl_2_ under the hydrothermal condition. In the centrosymmetric title complex, the coordination sphere of each Hg^II^ ion can be described as a distorted square pyramid, as indicated by the τ value of 0.33 (Addison *et al.*, 1984). The Hg^II^ ion coordinates to one terminal chloride ion, two bridging chloride ions and two chelating nitrogen donors from the same 2-bpt ligand (Fig. 1). Each 2-bpt ligand adopts the anti-conformation considering its two terminal pyridyl nitrogen, with the bidentate chelating coordination to one Hg^II^ center. In addition, the two adjacent Hg^II^ centers are linked by a pair of chloride bridges to form a Hg_2_Cl_2_ subunit, in which the Hg···Hg separation is 3.856 (1) Å and the Hg–Cl–Hg angle is 93.22 (3)°. There is an intramolecular N5–H5B···N6 hydrogen bond in the complex (Table 1).

In the crystal the binuclear units are connected to form a two-dimensional supramolecular network *via* intermolecular N-H···Cl hydrogen bonds (Table 1 and Fig. 2). In addition, π–π stacking interactions are present and further reinforce the two-dimensional supramolecular network. The centroid-centroid distance of the involved pyridyl rings, (N1/C1—C5) and (N6/C8—C12)^i^, is 3.696 (2) Å, while the centroid-centroid distance involving the triazole rings, (N2—N4/C6,C7) and (N2—N4/C6,C7)^i^, is 3.526 (2) Å (Fig. 2; symmetry code: (i) = -x+2, -y+1, -z+2).

### Experimental

A mixture of 2-bpt (23.8 mg, 0.1 mmol), HgCl_2_ (27.1 mg, 0.1 mmol) in water (10 ml) was sealed in a Teflon-lined stainless steel vessel (20 ml), which was heated to 413 K over a period of 24 h. It was then gradually cooled to room temperature at a rate of 5 °C/h. Colourless block-like crystals, suitable for X-ray analysis, were obtained. Anal. Calc. for C_24_H_20_Cl_4_Hg_2_N_12_: C, 28.27; H, 1.98; N, 16.49%. Found: C, 28.30; H, 1.94; N, 16.47%.

### Refinement

All the H-atoms were initially located in a difference Fourier map. The C—H and N—H atoms were then constrained to an ideal geometry, and refined as riding atoms: C—H = 0.93 Å and N—H = 0.89 Å, with *U*_iso_(H) = 1.2*U*_eq_(C) and *U*_iso_(H) = 1.5*U*_eq_(N).

### Crystal data

**Table:**

[Hg_2_Cl_4_(C_12_H_10_N_6_)_2_]	*F*(000) = 1904
*M**_r_* = 1019.50	*D*_x_ = 2.306 Mg m^−^^3^
Orthorhombic, *P**b**c**a*	Mo *K*α radiation, λ = 0.71073 Å
Hall symbol: -P 2ac 2ab	Cell parameters from 5032 reflections
*a* = 11.3634 (4) Å	θ = 2.5–28.1°
*b* = 14.9962 (6) Å	µ = 10.85 mm^−^^1^
*c* = 17.2328 (7) Å	*T* = 296 K
*V* = 2936.6 (2) Å^3^	Block, colourless
*Z* = 4	0.28 × 0.22 × 0.20 mm

### Data collection

**Table:**

Bruker SMART CCD area-detector diffractometer	2596 independent reflections
Radiation source: fine-focus sealed tube	2071 reflections with *I* > 2σ(*I*)
graphite	*R*_int_ = 0.023
phi and ω scans	θ_max_ = 25.0°, θ_min_ = 2.4°
Absorption correction: multi-scan (*SADABS*; Sheldrick, 1996)	*h* = −8→13
*T*_min_ = 0.475, *T*_max_ = 1.000	*k* = −16→17
14063 measured reflections	*l* = −20→19

### Refinement

**Table:**

Refinement on *F*^2^	Primary atom site location: structure-invariant direct methods
Least-squares matrix: full	Secondary atom site location: difference Fourier map
*R*[*F*^2^ > 2σ(*F*^2^)] = 0.022	Hydrogen site location: inferred from neighbouring sites
*wR*(*F*^2^) = 0.058	H-atom parameters constrained
*S* = 1.08	*w* = 1/[σ^2^(*F*_o_^2^) + (0.028*P*)^2^ + 2.8778*P*] where *P* = (*F*_o_^2^ + 2*F*_c_^2^)/3
2596 reflections	(Δ/σ)_max_ = 0.001
190 parameters	Δρ_max_ = 0.53 e Å^−^^3^
0 restraints	Δρ_min_ = −1.26 e Å^−^^3^

### Special details

**Table:**

Geometry. All e.s.d.'s (except the e.s.d. in the dihedral angle between two l.s. planes) are estimated using the full covariance matrix. The cell e.s.d.'s are taken into account individually in the estimation of e.s.d.'s in distances, angles and torsion angles; correlations between e.s.d.'s in cell parameters are only used when they are defined by crystal symmetry. An approximate (isotropic) treatment of cell e.s.d.'s is used for estimating e.s.d.'s involving l.s. planes.
Refinement. Refinement of *F*^2^ against ALL reflections. The weighted *R*-factor *wR* and goodness of fit *S* are based on *F*^2^, conventional *R*-factors *R* are based on *F*, with *F* set to zero for negative *F*^2^. The threshold expression of *F*^2^ > σ(*F*^2^) is used only for calculating *R*-factors(gt) *etc*. and is not relevant to the choice of reflections for refinement. *R*-factors based on *F*^2^ are statistically about twice as large as those based on *F*, and *R*- factors based on ALL data will be even larger.

### Fractional atomic coordinates and isotropic or equivalent isotropic displacement parameters (Å^2^)

**Table:**

	*x*	*y*	*z*	*U*_iso_*/*U*_eq_	
Hg1	0.661120 (15)	0.459756 (11)	0.997612 (9)	0.04268 (8)	
Cl1	0.66817 (10)	0.31955 (7)	0.92929 (6)	0.0460 (3)	
Cl2	0.53708 (10)	0.57586 (7)	0.92105 (6)	0.0486 (3)	
N1	0.7468 (3)	0.5214 (2)	1.11219 (19)	0.0384 (8)	
N2	0.8392 (3)	0.5359 (2)	0.9665 (2)	0.0381 (9)	
N3	0.8938 (3)	0.5633 (2)	0.89923 (19)	0.0377 (8)	
N4	0.9726 (3)	0.63366 (19)	0.99779 (15)	0.0266 (7)	
N5	1.0319 (3)	0.6981 (2)	1.04316 (18)	0.0356 (8)	
H5A	1.0021	0.7495	1.0264	0.053*	
H5B	1.1086	0.6932	1.0335	0.053*	
N6	1.1599 (3)	0.6863 (2)	0.89418 (19)	0.0413 (9)	
C1	0.9194 (4)	0.5833 (3)	1.1697 (2)	0.0377 (10)	
H1	0.9916	0.6116	1.1636	0.045*	
C2	0.8783 (5)	0.5612 (3)	1.2427 (2)	0.0489 (12)	
H2	0.9231	0.5735	1.2866	0.059*	
C3	0.7707 (5)	0.5210 (3)	1.2493 (3)	0.0554 (13)	
H3	0.7406	0.5068	1.2980	0.066*	
C4	0.7081 (4)	0.5017 (3)	1.1836 (3)	0.0504 (12)	
H4	0.6354	0.4738	1.1888	0.060*	
C5	0.8509 (4)	0.5626 (3)	1.1058 (2)	0.0326 (9)	
C6	0.8872 (4)	0.5792 (3)	1.0255 (2)	0.0292 (8)	
C7	0.9726 (3)	0.6220 (2)	0.9190 (2)	0.0286 (8)	
C8	1.0551 (3)	0.6651 (2)	0.8653 (2)	0.0300 (9)	
C9	1.0248 (4)	0.6771 (3)	0.7884 (2)	0.0438 (10)	
H9	0.9512	0.6599	0.7702	0.053*	
C10	1.1062 (5)	0.7153 (3)	0.7393 (2)	0.0506 (13)	
H10	1.0883	0.7250	0.6873	0.061*	
C11	1.2143 (5)	0.7389 (3)	0.7688 (3)	0.0536 (13)	
H11	1.2710	0.7650	0.7371	0.064*	
C12	1.2368 (4)	0.7232 (3)	0.8453 (3)	0.0515 (12)	
H12	1.3102	0.7392	0.8646	0.062*	

### Atomic displacement parameters (Å^2^)

**Table:**

	*U*^11^	*U*^22^	*U*^33^	*U*^12^	*U*^13^	*U*^23^
Hg1	0.04114 (12)	0.03899 (12)	0.04791 (13)	−0.00931 (7)	0.00559 (8)	−0.00763 (7)
Cl1	0.0512 (7)	0.0409 (6)	0.0458 (6)	−0.0077 (5)	0.0005 (5)	−0.0119 (5)
Cl2	0.0474 (7)	0.0458 (6)	0.0524 (7)	0.0020 (5)	0.0129 (5)	0.0116 (5)
N1	0.038 (2)	0.0356 (19)	0.0413 (19)	−0.0031 (16)	0.0071 (16)	0.0029 (15)
N2	0.039 (2)	0.039 (2)	0.0355 (19)	−0.0087 (17)	0.0010 (16)	−0.0032 (16)
N3	0.041 (2)	0.040 (2)	0.0321 (18)	−0.0088 (17)	0.0007 (16)	−0.0049 (15)
N4	0.0283 (17)	0.0246 (16)	0.0271 (16)	−0.0013 (13)	−0.0005 (14)	−0.0011 (13)
N5	0.042 (2)	0.0365 (19)	0.0283 (17)	−0.0086 (16)	−0.0004 (16)	−0.0082 (14)
N6	0.042 (2)	0.048 (2)	0.0347 (18)	−0.0137 (17)	0.0009 (16)	0.0035 (16)
C1	0.043 (3)	0.037 (2)	0.033 (2)	0.0008 (19)	0.0031 (19)	0.0026 (18)
C2	0.063 (3)	0.049 (3)	0.035 (2)	0.005 (3)	0.004 (2)	0.004 (2)
C3	0.073 (4)	0.053 (3)	0.040 (3)	0.002 (3)	0.021 (3)	0.008 (2)
C4	0.051 (3)	0.047 (3)	0.054 (3)	−0.007 (2)	0.017 (2)	0.009 (2)
C5	0.036 (2)	0.024 (2)	0.038 (2)	0.0060 (18)	0.0041 (18)	0.0025 (17)
C6	0.030 (2)	0.025 (2)	0.0319 (19)	0.0008 (17)	0.0031 (17)	−0.0020 (16)
C7	0.033 (2)	0.0270 (19)	0.0261 (18)	0.0032 (17)	−0.0026 (17)	−0.0008 (15)
C8	0.037 (2)	0.028 (2)	0.0251 (19)	0.0000 (18)	0.0040 (17)	−0.0004 (15)
C9	0.052 (3)	0.047 (3)	0.032 (2)	−0.002 (2)	−0.007 (2)	−0.0006 (18)
C10	0.085 (4)	0.041 (2)	0.026 (2)	0.003 (3)	0.007 (2)	0.0065 (18)
C11	0.072 (4)	0.045 (3)	0.044 (3)	−0.011 (3)	0.017 (3)	0.004 (2)
C12	0.050 (3)	0.051 (3)	0.054 (3)	−0.018 (2)	0.005 (2)	0.002 (2)

### Geometric parameters (Å, °)

**Table:**

Hg1—N2	2.385 (3)	C1—C2	1.383 (6)
Hg1—N1	2.388 (3)	C1—C5	1.384 (6)
Hg1—Cl1	2.4111 (10)	C1—H1	0.9300
Hg1—Cl2	2.5998 (11)	C2—C3	1.368 (7)
Hg1—Cl2^i^	2.7061 (11)	C2—H2	0.9300
Cl2—Hg1^i^	2.7061 (11)	C3—C4	1.368 (7)
N1—C5	1.339 (5)	C3—H3	0.9300
N1—C4	1.340 (5)	C4—H4	0.9300
N2—C6	1.324 (5)	C5—C6	1.465 (5)
N2—N3	1.378 (5)	C7—C8	1.468 (5)
N3—C7	1.301 (5)	C8—C9	1.380 (5)
N4—C6	1.355 (5)	C9—C10	1.378 (6)
N4—C7	1.368 (4)	C9—H9	0.9300
N4—N5	1.414 (4)	C10—C11	1.376 (8)
N5—H5A	0.8901	C10—H10	0.9300
N5—H5B	0.8900	C11—C12	1.365 (6)
N6—C8	1.329 (5)	C11—H11	0.9300
N6—C12	1.333 (5)	C12—H12	0.9300
			
N2—Hg1—N1	69.78 (12)	C2—C3—C4	119.3 (4)
N2—Hg1—Cl1	106.24 (9)	C2—C3—H3	120.4
N1—Hg1—Cl1	136.70 (9)	C4—C3—H3	120.4
N2—Hg1—Cl2	91.45 (9)	N1—C4—C3	122.9 (5)
N1—Hg1—Cl2	112.43 (8)	N1—C4—H4	118.6
Cl1—Hg1—Cl2	110.74 (4)	C3—C4—H4	118.6
N2—Hg1—Cl2^i^	156.52 (9)	N1—C5—C1	122.3 (4)
N1—Hg1—Cl2^i^	89.27 (8)	N1—C5—C6	113.9 (4)
Cl1—Hg1—Cl2^i^	96.23 (4)	C1—C5—C6	123.7 (4)
Cl2—Hg1—Cl2^i^	86.78 (3)	N2—C6—N4	108.6 (3)
Hg1—Cl2—Hg1^i^	93.22 (3)	N2—C6—C5	121.7 (4)
C5—N1—C4	117.9 (4)	N4—C6—C5	129.6 (4)
C5—N1—Hg1	118.1 (3)	N3—C7—N4	110.3 (3)
C4—N1—Hg1	122.7 (3)	N3—C7—C8	124.9 (3)
C6—N2—N3	108.4 (3)	N4—C7—C8	124.8 (3)
C6—N2—Hg1	114.3 (3)	N6—C8—C9	123.5 (4)
N3—N2—Hg1	135.5 (3)	N6—C8—C7	116.2 (3)
C7—N3—N2	106.9 (3)	C9—C8—C7	120.2 (4)
C6—N4—C7	105.9 (3)	C10—C9—C8	118.4 (4)
C6—N4—N5	123.9 (3)	C10—C9—H9	120.8
C7—N4—N5	129.4 (3)	C8—C9—H9	120.8
N4—N5—H5A	103.4	C11—C10—C9	118.7 (4)
N4—N5—H5B	107.9	C11—C10—H10	120.6
H5A—N5—H5B	112.5	C9—C10—H10	120.6
C8—N6—C12	116.7 (4)	C12—C11—C10	118.6 (4)
C2—C1—C5	118.7 (4)	C12—C11—H11	120.7
C2—C1—H1	120.6	C10—C11—H11	120.7
C5—C1—H1	120.6	N6—C12—C11	124.1 (5)
C3—C2—C1	118.9 (4)	N6—C12—H12	118.0
C3—C2—H2	120.6	C11—C12—H12	118.0
C1—C2—H2	120.6		
			
N2—Hg1—Cl2—Hg1^i^	−156.57 (9)	C2—C1—C5—C6	−177.3 (4)
N1—Hg1—Cl2—Hg1^i^	−87.88 (9)	N3—N2—C6—N4	−0.5 (4)
Cl1—Hg1—Cl2—Hg1^i^	95.44 (4)	Hg1—N2—C6—N4	−167.7 (2)
Cl2^i^—Hg1—Cl2—Hg1^i^	0.0	N3—N2—C6—C5	−177.9 (4)
N2—Hg1—N1—C5	−3.4 (3)	Hg1—N2—C6—C5	14.9 (5)
Cl1—Hg1—N1—C5	89.1 (3)	C7—N4—C6—N2	0.9 (4)
Cl2—Hg1—N1—C5	−86.3 (3)	N5—N4—C6—N2	171.6 (3)
Cl2^i^—Hg1—N1—C5	−172.6 (3)	C7—N4—C6—C5	178.0 (4)
N2—Hg1—N1—C4	−169.9 (4)	N5—N4—C6—C5	−11.2 (6)
Cl1—Hg1—N1—C4	−77.4 (4)	N1—C5—C6—N2	−17.9 (5)
Cl2—Hg1—N1—C4	107.1 (3)	C1—C5—C6—N2	159.1 (4)
Cl2^i^—Hg1—N1—C4	20.9 (3)	N1—C5—C6—N4	165.3 (4)
N1—Hg1—N2—C6	−5.9 (3)	C1—C5—C6—N4	−17.8 (7)
Cl1—Hg1—N2—C6	−140.4 (3)	N2—N3—C7—N4	0.6 (4)
Cl2—Hg1—N2—C6	107.5 (3)	N2—N3—C7—C8	176.7 (3)
Cl2^i^—Hg1—N2—C6	22.2 (4)	C6—N4—C7—N3	−0.9 (4)
N1—Hg1—N2—N3	−168.5 (4)	N5—N4—C7—N3	−171.0 (4)
Cl1—Hg1—N2—N3	57.0 (4)	C6—N4—C7—C8	−177.0 (3)
Cl2—Hg1—N2—N3	−55.1 (4)	N5—N4—C7—C8	12.9 (6)
Cl2^i^—Hg1—N2—N3	−140.3 (3)	C12—N6—C8—C9	1.8 (6)
C6—N2—N3—C7	−0.1 (4)	C12—N6—C8—C7	178.2 (4)
Hg1—N2—N3—C7	163.2 (3)	N3—C7—C8—N6	−148.0 (4)
C5—C1—C2—C3	−1.0 (6)	N4—C7—C8—N6	27.6 (5)
C1—C2—C3—C4	1.6 (7)	N3—C7—C8—C9	28.6 (6)
C5—N1—C4—C3	−0.9 (7)	N4—C7—C8—C9	−155.9 (4)
Hg1—N1—C4—C3	165.7 (4)	N6—C8—C9—C10	−1.8 (6)
C2—C3—C4—N1	−0.6 (7)	C7—C8—C9—C10	−178.1 (4)
C4—N1—C5—C1	1.5 (6)	C8—C9—C10—C11	0.7 (7)
Hg1—N1—C5—C1	−165.7 (3)	C9—C10—C11—C12	0.2 (7)
C4—N1—C5—C6	178.5 (4)	C8—N6—C12—C11	−0.8 (7)
Hg1—N1—C5—C6	11.3 (4)	C10—C11—C12—N6	−0.2 (7)
C2—C1—C5—N1	−0.5 (6)		

Symmetry codes: (i) −*x*+1, −*y*+1, −*z*+2.

### Hydrogen-bond geometry (Å, °)

**Table:**

*D*—H···*A*	*D*—H	H···*A*	*D*···*A*	*D*—H···*A*
N5—H5A···Cl2^ii^	0.89	2.80	3.446 (3)	131
N5—H5A···Cl1^iii^	0.89	2.77	3.512 (4)	142
N5—H5B···Cl1^iv^	0.89	2.62	3.452 (4)	155
N5—H5B···N6	0.89	2.47	2.956 (5)	115

Symmetry codes: (ii) *x*+1/2, −*y*+3/2, −*z*+2; (iii) −*x*+3/2, *y*+1/2, *z*; (iv) −*x*+2, −*y*+1, −*z*+2.
